# A case of dissociative identity disorder and attention deficit hyperactivity disorder comorbidity

**DOI:** 10.1192/j.eurpsy.2022.1196

**Published:** 2022-09-01

**Authors:** E.F. Aydın, T. Koca Laçin

**Affiliations:** University of Atatürk, Department Of Mental Health And Diseases, Erzurum, Turkey

**Keywords:** disorder comorbidity, Dissociative Identity disorder, Attention Deficit Hyperactivity Disorder, methylphenidate

## Abstract

**Introduction:**

Dissociative identity disorder(DID) is characterized by the existence of two or more distinct identities which involve changes in consciousness, emotion, memory, and behavior. It is associated with childhood traumatic experiences and other psychiatric disorders. Comorbidity in DID can lead to complex clinical presentations, poor treatment responses. Thus, it is crucial to identify patients with comorbidity and take them into the treatment plan.

**Objectives:**

We aim to report a case of DID and Attention-Deficit/Hyperactivity Disorder(ADHD) comorbidity.

**Methods:**

A case report is presented alongside a review of the relevant literature regarding “dissociative identity disorder” and “attention deficit hyperactivity disorder”.

**Results:**

We describe the case of a 39-year-old woman with DID, onsetting at age 25, who had consistently responded poorly to long-term psychotherapy and pharmacological treatment. She presented with anxiety, distinct personality states, alterations in memory, consciousness and behavior problems in functioning, and high Dissociative Experiences Scale(DES) scores. Throughout the interviews, we noticed that she had limited attention, excess movements. After a detailed evaluation, diagnosis of ADHD is established, using the Diagnostic Interview for ADHD(DIVA) and ADHD Self-Reporting Scale(ASRS). Methylphenidate was prescribed in addition to previous medication. İmprovement in the severity of both ADHD and DID symptoms was presented with lower scores in DES and ASRS after the introduction of methylphenidate with progressive dose adjusting till 60mg/day.

**Conclusions:**

Although previous studies demonstrated ADHD symptoms are related to dissociation, there is no well-established strategy for this. We believe that this case report provides a better approach to the comorbidity of ADHD and DID.

**Disclosure:**

No significant relationships.

